# Comparison of prasugrel and clopidogrel reloading on high platelet reactivity in clopidogrel-loaded patients undergoing percutaneous coronary intervention (PRAISE-HPR): a study protocol for a prospective randomized controlled clinical trial

**DOI:** 10.1186/1745-6215-14-62

**Published:** 2013-02-28

**Authors:** Dong-Hyun Lee, Moo Hyun Kim, Tae-Ho Park, Jong Sung Park, Kyungil Park, Hong-Zhe Zhang, Jeong-Min Seo, Michael S Lee

**Affiliations:** 1 Department of Cardiology, Dong-A University Hospital, 3-1, Dongdaeshin-Dong, Seo-Gu, Busan 602-715, Republic of Korea; 2 Regional Clinical Trial Center, Dong-A University Hospital, 3-1, Dongdaeshin-Dong, Seo-Gu, Busan 602-715, Republic of Korea; 3 UCLA Medical Center, 1271 Stoner Ave # 409, Los Angeles, CA, 90025, USA

**Keywords:** Prasugrel, Clopidogrel, Acute coronary syndrome, Platelet reactivity

## Abstract

**Background:**

Patients with reduced responsiveness to clopidogrel often have diminished platelet inhibition, a factor associated with increased rates of major adverse cardiovascular events. Clinical trials that have focused on reducing high on-treatment platelet reactivity (HPR) with an additional loading dose of clopidogrel have reported varying effects. Prasugrel, a newer thienopyridine, exhibits a more consistent antiplatelet effect and more rapid onset time when compared to clopidogrel. We hypothesize that prasugrel reloading would be more effective than clopidogrel reloading in patients with HPR after an initial loading dose of clopidogrel.

**Method/Design:**

Comparison of Prasugrel and Clopidogrel Reloading on High Platelet Reactivity in Clopidogrel-loaded Patients Undergoing Percutaneous Coronary Intervention (PRAISE-HPR) is a prospective, randomized, open-label, active controlled study. A total of 76 patients undergoing percutaneous coronary intervention (PCI), with HPR after administration of a loading dose of clopidogrel will be randomly assigned to either prasugrel or clopidogrel groups, and patients in each group will be reloaded with 20 mg of prasugrel or 300 mg of clopidogrel. The primary endpoint will be HPR at 24 hours after PCI, as determined by the VerifyNow assay during the study period. The rate of sustained high platelet reactivity and 30-day clinical outcomes will also be measured.

**Discussion:**

PRAISE-HPR is a randomized controlled clinical trial to investigate the efficacy and safety of reloading prasugrel and clopidogrel in suppressing residual high platelet reactivity. The results will be made publicly available in the year 2013.

**Trial registration:**

NCT01609647

## Background

In patients with acute coronary syndrome (ACS) who undergo percutaneous coronary interventions (PCI), common recommendations for antithrombotic regimens include dual antiplatelet therapy with aspirin and clopidogrel [1,2]. Clopidogrel is a thienopyridine derivative metabolized by cytochrome P450 to an active metabolite that irreversibly inhibits platelet P2Y_12_ receptors [3]. Although the efficacy of clopidogrel has been well established, it exhibits pharmacodynamic variability in diverse conditions which can be due to the loss-of-function *CYP2C19* allele, drug-drug interactions or clinical factors [4]. High on-treatment platelet reactivity (HPR) after clopidogrel administration is associated with adverse cardiovascular events [5-11] and the rate of HPR can be reduced with either a higher loading dose (LD) or maintenance doses (MD) of clopidogrel, or a potent alternative drug [2,12-14]. The use of double doses of clopidogrel and the more potent thienopyridine prasugrel to reduce HPR and subsequent cardiovascular events have not been successful, partially due to the studies being conducted with low event rates and low-risk patients [15,16]. Another issue concerning prior study design has been the delayed administration of drugs, administered after PCI in both cases. Therefore, a platelet reactivity (PR) guided trial with reloading of a more potent drug in the early phase of ACS, especially before PCI, is warranted.

Prasugrel is also a thienopyridine derivative. However, the metabolism of prasugrel is much more efficient than that of clopidogrel, because it occurs via esterases and has less dependency on cytochrome P450 enzymes [17]. As a result, prasugrel treatment results in more rapid onset, more consistent conversion from the inactive prodrug to active metabolite, and higher blood concentrations of the active metabolite [18]. A LD of prasugrel 60 mg results in more rapid, potent and consistent inhibition of platelet function than clopidogrel LDs of 300 mg or 600 mg [19].

We hypothesize that for patients with HPR after an initial LD of clopidogrel, reloading with prasugrel in the early phase of ACS will reduce PR more effectively than reloading with clopidogrel, resulting in a reduction of adverse cardiovascular events.

## Methods and design

### Trial objectives

The primary aim of this study is to compare PR if 20 mg of prasugrel is reloaded, followed by MDs of 5 mg daily, versus clopidogrel reloading with 300 mg followed by MDs of 75mg per day, prior to PCI in ACS patients with HPR after an initial LD of clopidogrel.

### Study design

**Figure 1:**
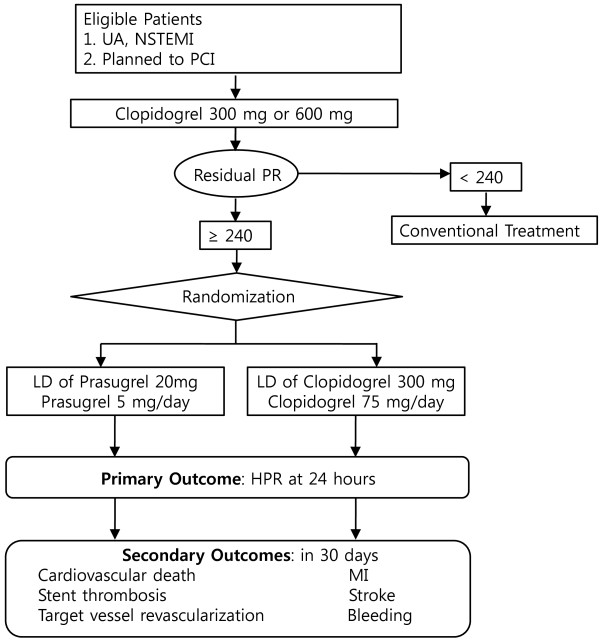
**Overall study design. **ACS, acute coronary syndrome; UA, unstable angina; NSTEMI, myocardial infarction without ST-segment elevation; PCI, percutaneous coronary intervention; PR, platelet reactivity; MI, myocardial infarction.

PRAISE-HPR is a prospective, randomized, open-label, active controlled study with two parallel study groups. It will be conducted at Dong-A University Hospital in Busan, Republic of Korea. Participants will be randomly allocated into prasugrel or clopidogrel reloading groups. Clinical endpoints will be measured at four weeks post-procedure. Recruitment of participants began in November 2012. The overall study design is depicted in Figure 1. Study approval was granted by the Institutional Review Board (IRB) of Dong-A University Hospital, and consecutive, eligible patients will be required to provide written informed consent.

### Randomization

Random treatment assignments will be generated using Excel spreadsheet software (Microsoft Corporation, Redmont, DC, USA). Eligible patients will be randomly assigned, on a 1:1 ratio, to receive reloading with either prasugrel or clopidogrel. Treatment randomization will be performed by independent personnel not involved in the study.

### Approval

This study will be conducted according to principles outlined in the Declaration of Helsinki. All patients must provide written informed consent, understanding that participation is voluntary and can be withdrawn at any time without any negative consequences concerning their current or future medical treatment. This study protocol has been approved by the IRB of Dong-A University Hospital.

### Patient population

Male and female patients, aged between 20 and 80 years for which the decision to undergo PCI within 24 hours has been made, due to ACS without ST-segment elevation are eligible for the study (Table 1).

**Table 1:** Eligibility criteria

**Inclusion criteria**	**Exclusion criteria**
Subjects 20 to 80 years old	Body weight < 50 kg
Male or female gender	Contraindications for study drugs
ACS without ST-segment elevation	Urgent PCI for ACS
Planned to undergo PCI	Use of glycoprotein IIb/IIIa inhibitor
Patients who provided written informed consent	History of transient ischemic attack
	Active internal bleeding or bleeding diathesis
	Upper gastrointestinal bleeding in the past six months
	Hemoglobin < 10 g/dl or platelets < 100,000/mm^3^
	Renal dysfunction (serum creatinine > 2.5 mg/dl)
	Hepatic dysfunction (serum transaminase > three times normal limit)

ACS, acute coronary syndrome; PCI, percutaneous coronary intervention.

Patients requiring urgent PCI for ACS will be excluded from entry, as will those with recent or planned medical treatment with glycoprotein IIb/IIIa inhibitors, body weight < 50 kg, contraindication to the study drug, a history of transient ischemic attack, active internal or bleeding diathesis, hemoglobin < 10 g/dl, platelet count < 100,000/mm^3^, renal insufficiency with serum creatinine level > 2.5 mg/dl or significant hepatic dysfunction.

### Intervention and comparator descriptions

Before randomization, 300 mg of aspirin and 600 mg of clopidogrel will be administered as LDs according to the conventional guidelines for ACS [2]. For those who have already been receiving clopidogrel for five days or more, 300 mg of clopidogrel will be administered as a LD. PR will be evaluated before PCI and defined as the time between the arrival of the patient at the angiography room, and the beginning of coronary angiography. Angiography will not begin before confirmation of PRU. Patients who exhibit HPR defined as a PRU value of 240 units or more by VerifyNow (Accumetrics, San Diego, CA, USA) will be randomized and followed by assignment to either the clopidogrel reloading group (clopidogrel 300 mg) or the prasugrel reloading group (prasugrel 20 mg). Angiography in patients with HPR will begin after administration of the study drug. PR will be checked serially using the VerifyNow (Accumetrics, San Diego, CA, USA) assay at four hours, 24 hours and 30 days after PCI. Patients will be required to visit the outpatient clinic at Day 30 for measurement of PR. For patients discharged before Day 8, a secondary visit at Day 15 will be recommended.

In the clopidogrel reloading group, 300 mg clopidogrel will be reloaded, followed by MDs of clopidogrel 75 mg/day for 30 days. In the prasugrel reloading group, 20 mg of prasugrel will be reloaded, followed by prasugrel administration of 5 mg/day for 30 days.

### Measurement of outcomes

The primary endpoint of this study will be HPR at 24 hours after PCI, as determined by the VerifyNow (Accumetrics, San Diego, CA, USA) assay during the study period (Table 2). The optimal cutoff value is defined as 240 units, as observed by Marcucci *et al*. [9]. Secondary endpoints include major adverse cardiovascular events at 30 days after PCI, including death from cardiovascular cause, myocardial infarction (MI), stent thrombosis, hemorrhagic or non-hemorrhagic stroke, and bleeding events defined by Thrombolysis in Myocardial Infarction (TIMI), as well as Bleeding Academic Research Consortium (BARC) criteria. HPR at four hours or 30 days after PCI will also be evaluated.

**Table 2:** Primary and secondary outcomes

**Primary outcome**	**Secondary outcomes**
HPR (PR ≥ 240 units)	Death from cardiovascular cause
	Myocardial infarction
	Stent thrombosis
	Hemorrhagic or non-hemorrhagic stroke
	Target vessel revascularization
	Bleeding event defined by Thrombolysis in Myocardial Infarction (TIMI) criteria and BARC

HPR, high platelet reactivity: more than 240 units determined via VerifyNow (Accumetrics, San Diego, CA, USA); PR, platelet reactivity; BARC, bleeding academic research consortium.

### Adverse effects

Patients will be interviewed daily during admission and at each visit after discharge regarding the occurrence of any adverse events, including time of onset, duration and severity. At each interview, patients will be assessed for reported bleeding classified by the TIMI bleeding classification scheme [20] as well as the BARC definition [21]. All information regarding adverse events will be to be presented on a case report form. The causal relation to the study drug and the intensity of adverse events will be evaluated by the investigators. Serious adverse events will be reported to the IRB and study sponsor by the principal investigator within 24 hours of being reported by the subjects or the treating physicians.

### Withdrawals

Participants are free to withdraw from trial participation at their own request at any time without giving reasons for their decisions. Additionally, the primary investigator may withdraw study participants if continuation of the trial is deemed to be detrimental to the patient’s well-being. Withdrawals will be documented in the case report form and in the patient’s medical records with active follow-up for ongoing severe adverse events. Distinction will be made between patients who ‘withdraw from the study’ versus ‘withdraw from the treatment’ in order to preserve the ability to analyze endpoints for all participants who underwent randomization, allowing for the later possibility of intention-to-treat inferences.

### Sample size

The sample size calculation is based on the primary outcome and the primary analysis for the intention-to-treat population. The sample size is calculated using the study’s primary objective to detect a 15% difference in PR suppression between reloading with prasugrel and clopidogrel, to a power of 80% to demonstrate difference. On the basis of previous reports we assume the inhibition of PR after a LD of clopidogrel to be approximately 55% of baseline [22]. The assumed inhibition of PR after LD of prasugrel is 70%. This was calculated from studies showing a 30% higher concentration of the active metabolite in patients of East Asian ethnicity [23] and an approximate 90% inhibition of baseline PRU two hours after a prasugrel 30 mg LD was given in healthy Korean people [24]. We expect the difference in PR inhibition will be at least 15% when considering ACS patients. We adjusted the sample size for an estimated follow-up loss rate of 20%, a two-sided level of significance of α = 5%, which determined that 38 patients in each group would be required to detect this difference with a two-sided Student’s *t* test. A total of 76 patients will be randomized and included in the analysis. We predict that 50% of patients will exhibit HPR as determined by VerifyNow (Accumetrics, San Diego, CA, USA) after receiving the clopidogrel LD. A total of 152 patients will be screened and 76 patients will be randomized and included in the analysis, with 38 patients in each group.

### Statistical analysis

The focus of this trial is to compare the efficacy and safety between both study groups. The statistical analyses will be performed on the full analysis set, which will include data from all randomized patients receiving at least one dose of study medication. The safety analysis will include calculation of frequencies, rates of complications, and occurrence of any serious adverse events reported in the two groups.

Descriptive statistics will be calculated according to the scale level of the variables. Continuous data will be analyzed using Student’s *t* test. The chi-square test will be used for categorical variables. A value of *P* < 0.05 will be used to denote statistical significance.

The effect of treatment will be expressed as relative risk estimates and absolute risk reduction. The primary outcome will be analyzed using multivariate logistic regression, adjusting for clinically relevant baseline imbalances. The remaining secondary outcomes will be analyzed using simple 2 × 2 tables and logistic regression. In all analyses, statistical uncertainty will be quantified via 95% confidence intervals. All statistical data will be analyzed using SPSS Version 12.0 (SPCC Inc., Chicago, IL, USA).

## Discussion

Although clopidogrel is an effective antiplatelet agent, variability in patient response and potential high PR remains a major shortfall. To date, recent studies focusing on tailored antiplatelet therapy have not shown benefits. However, a study comparing the efficacy of prasugrel and clopidogrel in early reloading for the management of HPR after clopidogrel LD has not been conducted. In addition, guidelines for managing HPR before or during PCI in ACS patients have not documented.

Our current investigation into reloading prasugrel versus clopidogrel for reductions of HPR in ACS patients undergoing PCI is based on the hypothesis that prasugrel reloading is superior to clopidogrel for HPR and adverse event reduction, including periprocedural MI. Our protocol design has some safeguards to avoid performance and evaluation bias. Randomization will be performed with computer software. Data will be recorded and analyzed by specialists who are not aware of treatment allocation.

A major safety concern for this trial is an increased risk of bleeding. In TRITON-TIMI 38, higher rates of bleeding (particularly non-coronary artery bypass graft-related TIMI major bleeding) were observed in the prasugrel group [25]. However, in comparison to clopidogrel, prasugrel provided greater protection from ischemic events (particularly stent thrombosis, urgent revascularization and MI). We therefore anticipate that the benefit of antiplatelet efficacy will outweigh the increased risk of bleeding.

Regarding the issue of loading and maintenance doses, previous reports have revealed that patients of East Asian ethnicity exhibit on average 30% higher levels of active metabolite compared to Caucasians, with the implication that exposure of mean doses of prasugrel to Asian subjects may not be appropriate [23]. Other studies focused on Chinese subjects have determined that 30 mg of prasugrel produces higher concentrations of active metabolite and greater platelet inhibition than 300 mg of clopidogrel [26]. Peak levels of prasugrel active metabolites were found to be 67% higher in Chinese subjects (calculated to be 39% higher after adjustment for differences in body weight) than in Caucasian volunteers. Taking into account ethnic differences in body weight and body mass index, subjects of North-East Asian ethnicity appear to receive comparable effects with lower prasugrel LDs and MDs. In an additional study conducted by our team, healthy Korean volunteers administered with a 30 mg LD of prasugrel exhibited significantly greater and more rapid inhibition of PR than those administered with a clopidogrel LD of 600 mg [24]. Yokoi *et al*. compared several different prasugrel LDs and MDs in Japanese patients and reported similar effects for a 10 mg LD/ 2.5mg MD prasugrel regimen to a 300mg LD/75 mg MD clopidogrel regimen [27]. These dosages are one sixth and one fourth, respectively, of the current LD and MD recommended by the manufacturer. We plan to conduct the present study with lower LDs and MDs of prasugrel than doses previously recommended.

Due to its rapid onset of action, the use of prasugrel can be particularly favorable in patients requiring PCI at the time of angiography who have not received thienopyridine loading. LDs of prasugrel can be expected to provide more potent platelet inhibition within a short period of time for PCI support. The use of prasugrel therefore appears particularly suitable for patients undergoing PCI within hours of the decision to use a thienopyridine. Furthermore, this rapid onset agent can be administered immediately prior to, or during PCI. This is particularly relevant for patients with HPR remaining after a LD of clopidogrel.

The GRAVITAS [16] and TRIGGER-PCI [15] trials aimed to detect reductions in HPR and concluded negative results. However, these studies did not use reloading or include high risk, acute phase patients. Our study is therefore the first to involve early manipulation of HPR using a more potent agent before or during PCI. The TRITON-TIMI 38 trial was noted for a lack of clopidogrel loading before randomization. Our trial design addresses some ethical issues arising from such protocols, due to closer similarities with real-world treatment. We anticipate that our study will reveal the possible role of prasugrel reloading and maintenance in improving clinical outcomes, emphasizing its potential to become an integral component in the management of patients undergoing PCI with remaining HPR after a loading dose of clopidogrel.

## Trial status

PRAISE-HPR began randomizing patients in November 2012. The principle investigator can be contacted by Email.

## Abbreviations

ACS: Acute coronary syndrome; PCI: Percutaneous coronary intervention; PR: Platelet reactivity; HPR: High on-treatment platelet reactivity; IRB: Institutional review board; LD: Loading dose; MD: Maintenance dose.

## Competing interests

The authors declare that they have no competing interests.

## Authors’ contributions

DHL contributed to the study design and to drafting and revising the manuscript. MHK, as a principle investigator, provided the concept of the study and reviewed the manuscript. THP, JSP, KP, HZ JMS and MSL actively contributed to the study design and all authors have read and approved the final manuscript.

## References

[B1] Yusuf S Zhao F Mehta SR Chrolavicius S Tognoni G Fox KK Effects of clopidogrel in addition to aspirin in patients with acute coronary syndromes without ST-segment elevation N Engl J Med 2001 345 494 502 11519503 10.1056/NEJMoa010746

[B2] King SB III Smith SC Jr Hirshfeld JW Jr Jacobs AK Morrison DA Williams DO Feldman TE Kern MJ O'Neill WW Schaff HV 2007 focused update of the ACC/AHA/SCAI 2005 guideline update for percutaneous coronary intervention: a report of the American College of Cardiology/American Heart Association Task Force on practice guidelines: 2007 writing group to review new evidence and update the ACC/AHA/SCAI 2005 guideline update for percutaneous coronary intervention, writing on behalf of the 2005 writing committee Circulation 2008 117 261 295 10.1161/CIRCULATIONAHA.107.188208 18079354

[B3] Savi P Herbert JM Clopidogrel and ticlopidine: P2Y12 adenosine diphosphate-receptor antagonists for the prevention of atherothrombosis Semin Thromb Hemost 2005 31 174 183 10.1055/s-2005-869523 15852221

[B4] Gurbel PA Bliden KP Hiatt BL O'Connor CM Clopidogrel for coronary stenting: response variability, drug resistance, and the effect of pretreatment platelet reactivity Circulation 2003 107 2908 2913 10.1161/01.CIR.0000072771.11429.83 12796140

[B5] Simon T Verstuyft C Mary-Krause M Quteineh L Drouet E Meneveau N Steg PG Ferrieres J Danchin N Becquemont L Genetic determinants of response to clopidogrel and cardiovascular events N Engl J Med 2009 360 363 375 10.1056/NEJMoa0808227 19106083

[B6] Matetzky S Shenkman B Guetta V Shechter M Beinart R Goldenberg I Novikov I Pres H Savion N Varon D Hod H Clopidogrel resistance is associated with increased risk of recurrent atherothrombotic events in patients with acute myocardial infarction Circulation 2004 109 3171 3175 10.1161/01.CIR.0000130846.46168.03 15184279

[B7] Gurbel PA Bliden KP Samara W Yoho JA Hayes K Fissha MZ Tantry US Clopidogrel effect on platelet reactivity in patients with stent thrombosis: results of the CREST Study J Am Coll Cardiol 2005 46 1827 1832 10.1016/j.jacc.2005.07.056 16286166

[B8] Hochholzer W Trenk D Bestehorn HP Fischer B Valina CM Ferenc M Gick M Caputo A Buttner HJ Neumann FJ Impact of the degree of peri-interventional platelet inhibition after loading with clopidogrel on early clinical outcome of elective coronary stent placement J Am Coll Cardiol 2006 48 1742 1750 10.1016/j.jacc.2006.06.065 17084243

[B9] Marcucci R Gori AM Paniccia R Giusti B Valente S Giglioli C Buonamici P Antoniucci D Abbate R Gensini GF Cardiovascular death and nonfatal myocardial infarction in acute coronary syndrome patients receiving coronary stenting are predicted by residual platelet reactivity to ADP detected by a point-of-care assay: a 12-month follow-up Circulation 2009 119 237 242 10.1161/CIRCULATIONAHA.108.812636 19118249

[B10] Patti G Nusca A Mangiacapra F Gatto L D'Ambrosio A Di Sciascio G Point-of-care measurement of clopidogrel responsiveness predicts clinical outcome in patients undergoing percutaneous coronary intervention results of the ARMYDA-PRO (Antiplatelet therapy for Reduction of MYocardial Damage during Angioplasty-Platelet Reactivity Predicts Outcome) study J Am Coll Cardiol 2008 52 1128 1133 10.1016/j.jacc.2008.06.038 18804738

[B11] Sibbing D Braun S Morath T Mehilli J Vogt W Schomig A Kastrati A von Beckerath N Platelet reactivity after clopidogrel treatment assessed with point-of-care analysis and early drug-eluting stent thrombosis J Am Coll Cardiol 2009 53 849 856 10.1016/j.jacc.2008.11.030 19264241

[B12] von Beckerath N Taubert D Pogatsa-Murray G Schomig E Kastrati A Schomig A Absorption, metabolization, and antiplatelet effects of 300 mg, 600 mg, and 900 mg loading doses of clopidogrel: results of the ISAR-CHOICE (Intracoronary Stenting and Antithrombotic Regimen: Choose between three High Oral doses for Immediate Clopidogrel Effect) trial Circulation 2005 112 2946 2950 16260639 10.1161/CIRCULATIONAHA.105.559088

[B13] Angiolillo DJ Saucedo JF Deraad R Frelinger AL Gurbel PA Costigan TM Jakubowski JA Ojeh CK Effron MB Increased platelet inhibition after switching from maintenance clopidogrel to prasugrel in patients with acute coronary syndromes: results of the SWAP (SWitching Anti Platelet) study J Am Coll Cardiol 2010 56 1017 1023 10.1016/j.jacc.2010.02.072 20846599

[B14] Jernberg T Payne CD Winters KJ Darstein C Brandt JT Jakubowski JA Naganuma H Siegbahn A Wallentin L Prasugrel achieves greater inhibition of platelet aggregation and a lower rate of non-responders compared with clopidogrel in aspirin-treated patients with stable coronary artery disease Eur Heart J 2006 27 1166 1173 16621870 10.1093/eurheartj/ehi877

[B15] Trenk D Stone GW Gawaz M Kastrati A Angiolillo DJ Muller U Richardt G Jakubowski JA Neumann FJ A randomized trial of prasugrel versus clopidogrel in patients with high platelet reactivity on clopidogrel after elective percutaneous coronary intervention with implantation of drug-eluting stents: results of the TRIGGER-PCI (Testing Platelet Reactivity In Patients Undergoing Elective Stent Placement on Clopidogrel to Guide Alternative Therapy with Prasugrel) study J Am Coll Cardiol 2012 59 2159 2164 10.1016/j.jacc.2012.02.026 22520250

[B16] Price MJ Angiolillo DJ Teirstein PS Lillie E Manoukian SV Berger PB Tanguay JF Cannon CP Topol EJ Platelet reactivity and cardiovascular outcomes after percutaneous coronary intervention: a time-dependent analysis of the Gauging Responsiveness with a VerifyNow P2Y12 assay: Impact on Thrombosis and Safety (GRAVITAS) trial Circulation 2011 124 1132 1137 10.1161/CIRCULATIONAHA.111.029165 21875913

[B17] Sugidachi A Ogawa T Kurihara A Hagihara K Jakubowski JA Hashimoto M Niitsu Y Asai F The greater in vivo antiplatelet effects of prasugrel as compared to clopidogrel reflect more efficient generation of its active metabolite with similar antiplatelet activity to that of clopidogrel's active metabolite J Thromb Haemost 2007 5 1545 1551 10.1111/j.1538-7836.2007.02598.x 17456192

[B18] Brandt JT Payne CD Wiviott SD Weerakkody G Farid NA Small DS Jakubowski JA Naganuma H Winters KJ A comparison of prasugrel and clopidogrel loading doses on platelet function: magnitude of platelet inhibition is related to active metabolite formation Am Heart J 2007 153 66 e69 16 10.1016/j.ahj.2006.10.010 17174640

[B19] Wiviott SD Trenk D Frelinger AL O'Donoghue M Neumann FJ Michelson AD Angiolillo DJ Hod H Montalescot G Miller DL Prasugrel compared with high loading- and maintenance-dose clopidogrel in patients with planned percutaneous coronary intervention: the prasugrel in comparison to clopidogrel for inhibition of platelet activation and aggregation-thrombolysis in myocardial infarction 44 trial Circulation 2007 116 2923 2932 10.1161/CIRCULATIONAHA.107.740324 18056526

[B20] Chesebro JH Knatterud G Roberts R Borer J Cohen LS Dalen J Dodge HT Francis CK Hillis D Ludbrook P Thrombolysis in Myocardial Infarction (TIMI) Trial. Phase I: a comparison between intravenous tissue plasminogen activator and intravenous streptokinase. Clinical findings through hospital discharge Circulation 1987 76 142 154 10.1161/01.CIR.76.1.142 3109764

[B21] Mehran R Rao SV Bhatt DL Gibson CM Caixeta A Eikelboom J Kaul S Wiviott SD Menon V Nikolsky E Standardized bleeding definitions for cardiovascular clinical trials: a consensus report from the Bleeding Academic Research Consortium Circulation 2011 123 2736 2747 10.1161/CIRCULATIONAHA.110.009449 21670242

[B22] Wallentin L P2Y(12) inhibitors: differences in properties and mechanisms of action and potential consequences for clinical use Eur Heart J 2009 30 1964 1977 10.1093/eurheartj/ehp296 19633016

[B23] Small DS Kothare P Yuen E Lachno DR Li YG Winters KJ Farid NA Ni L Jakubowski JA Salazar DE The pharmacokinetics and pharmacodynamics of prasugrel in healthy Chinese, Japanese, and Korean subjects compared with healthy Caucasian subjects Eur J Clin Pharmacol 2010 66 127 135 10.1007/s00228-009-0737-1 19888568

[B24] Kim MH Zhang H-Z Jung DK Pharmacodynamic comparisons for single loading doses of prasugrel (30 mg) and clopidogrel (600 mg) in healthy Korean volunteers Circ Jin press 10.1253/circj.cj-12-0783 23363643

[B25] Wiviott SD Antman EM Gibson CM Montalescot G Riesmeyer J Weerakkody G Winters KJ Warmke JW McCabe CH Braunwald E Evaluation of prasugrel compared with clopidogrel in patients with acute coronary syndromes: design and rationale for the TRial to assess Improvement in Therapeutic Outcomes by optimizing platelet inhibitioN with prasugrel Thrombolysis In Myocardial Infarction 38 (TRITON-TIMI 38) Am Heart J 2006 152 627 635 10.1016/j.ahj.2006.04.012 16996826

[B26] Small DS Payne CD Kothare P Yuen E Natanegara F Teng Loh M Jakubowski JA Richard Lachno D Li YG Winters KJ Pharmacodynamics and pharmacokinetics of single doses of prasugrel 30 mg and clopidogrel 300 mg in healthy Chinese and white volunteers: an open-label trial Clin Ther 2010 32 365 379 10.1016/j.clinthera.2010.02.015 20206794

[B27] Yokoi H Kimura T Isshiki T Ogawa H Ikeda Y Pharmacodynamic assessment of a novel P2Y12 receptor antagonist in Japanese patients with coronary artery disease undergoing elective percutaneous coronary intervention Thromb Res 2012 129 623 628 10.1016/j.thromres.2011.11.023 22178576

